# Antimicrobial Resistance in European Companion Animals Practice: A One Health Approach

**DOI:** 10.3390/ani15121708

**Published:** 2025-06-09

**Authors:** Helena I. G. Monteiro, Vanessa Silva, Telma de Sousa, Rita Calouro, Sónia Saraiva, Gilberto Igrejas, Patrícia Poeta

**Affiliations:** 1 MicroART-Antibiotic Resistance Team, Department of Veterinary Sciences, University of Trás-os-Montes and Alto Douro, 5000-801 Vila Real, Portugal; al2024107632@alunos.utad.pt (H.I.G.M.); vanessasilva@utad.pt (V.S.); telmaslsousa@hotmail.com (T.d.S.); ritasousacalouro@gmail.com (R.C.); 2 Department of Genetics and Biotechnology, University of Trás-os-Montes and Alto Douro, 5000-801 Vila Real, Portugal; gigrejas@utad.pt; 3 Functional Genomics and Proteomics Unit, University of Trás-os-Montes and Alto Douro, 5000-801 Vila Real, Portugal; 4 Associated Laboratory for Green Chemistry, University NOVA of Lisbon, 1099-085 Caparica, Portugal; 5 Polytechnic Institute of Santarém, School of Agriculture, Quinta do Galinheiro, 2001-904 Santarém, Portugal; 6 Veterinary and Animal Research Centre, Associate Laboratory for Animal and Veterinary Science (AL4AnimalS), University of Trás-os-Montes and Alto Douro, 5000-801 Vila Real, Portugal; soniasaraiva@utad.pt; 7 CECAV—Veterinary and Animal Research Centre, University of Trás-os-Montes and Alto Douro, 5000-801 Vila Real, Portugal; 8 Department of Veterinary Sciences, School of Agricultural and Veterinary Sciences, University of Trás-os-Montes e Alto Douro, 5000-801 Vila Real, Portugal

**Keywords:** veterinary medicine, antibiotics, antibiotic resistance, companion animals, humans, One Health

## Abstract

Antimicrobial resistance (AMR) is a global health emergency. The growing levels of multidrug-resistant bacteria in companion animals represent a critical but under-recognized threat, as pets can act as reservoirs and vectors of resistant pathogens, and bacteria can spread between species. Despite living in close proximity to humans and sharing many of the same antibiotic classes, companion animals remain largely overlooked in the AMR control strategies. A major obstacle to combating AMR in Europe is the absence of a mandatory and uniform surveillance system. Without a standardized approach, data collection remains inconsistent, leading to knowledge gaps and hindering effective investigation and policymaking. Surveillance failures prevent a comprehensive understanding of resistance patterns, making it difficult to implement timely and evidence-based measures. Existing laws and regulations are insufficient to contain the growing threat of AMR. Current policies fail to enforce strict antimicrobial stewardship across all European countries and lack the necessary scope to address resistance effectively. Urgent action is required—Europe must implement stronger, harmonized surveillance systems, enforce stricter veterinary antimicrobial regulations, and develop more active and binding programs to curb resistance before it reaches an uncontrollable level. A One Health approach, recognizing the interconnectedness of human, animal, and environmental health, is essential.

## Simple Summary

Antimicrobial resistance is a significant global public health concern, and the role of companion animals in its spread cannot be overlooked. Sharing many of the same antibiotic classes as humans and living in close proximity to them, these animals present a serious risk of the transmission of antimicrobial-resistant bacteria and resistance genes between species. Resistance proportions have reached concerning levels, including up to 50% in cats in Denmark and 40% in dogs in France. However, current EU policies remain insufficient to address this situation. The absence of a mandatory, harmonized surveillance system across Europe has created a significant gap in the data and understanding, hindering effective monitoring and control efforts. The growing emergence of multidrug-resistant strains in small animals further highlights the urgent need for targeted veterinary interventions. To address this problem effectively, the EU must adopt stronger, more unified policies within a comprehensive One Health framework that recognizes the interconnectedness of human, animal, and environmental health.

## 1. Introduction

Antimicrobial resistance (AMR) is one of the most significant health threats globally, affecting both humans and animals, with antimicrobial use being the primary contributing factor [1,2,3,4]. Discussing antimicrobial resistance in companion animals is inseparable from addressing resistance in production animals, exotic and wildlife species, aquatic animals, the environment, and humans, as all these elements are interconnected. The population of companion animals has grown significantly in industrialized countries over the past few decades, with there being an estimated 79 million cats and 68 million dogs in 2023, and this trend is expected to continue [2,5,6]. As people and their companion animals share living spaces, creating conditions for the transmission of bacteria between species, they also share medications, particularly antibiotics, with several antibiotic classes used in both populations [7]. This overlap and the transmission of resistant bacteria highlight the potential for antibiotic use in companion animals to drive AMR. Given the implications of the presence of AMR bacteria in companion animals for human health, and the risks of infection with multidrug-resistant bacteria having dire consequences, this issue requires increased attention and stronger policies [2,8,9].

AMR in animals can be classified into three categories: AMR associated with specific animal pathogens, AMR in zoonotic pathogens, and AMR in commensal bacteria. Among these, commensal bacteria are of particular concern due to their greater biomass compared to the other groups [8,10]. Currently, several microorganisms are involved in critical scenarios of antimicrobial resistance, including methicillin-resistant *Staphylococcus pseudointermedius* (MRSP), methicillin-resistant *Staphylococcus aureus* (MRSA), and multidrug-resistant (MDR) Gram-negative bacteria, such as carbapenem-resistant *Acinetobacter baumannii* and *Enterobacteriaceae*. These microorganisms are commonly observed in intensive care settings but are increasingly spreading beyond healthcare facilities. With their high transmission capacity, they pose a significant risk to human and animal health [8,11,12].

Addressing this crisis demands a unified and cross-sectoral approach that transcends disciplinary barriers. A shared objective must be defined with stakeholders, ensuring data harmonization without compromising key goals [13]. Once vulnerabilities are identified, actions must follow. This includes enforcing stricter policies, strengthening legislation, enhancing oversight, regulating prescriptions through official channels (controlling both the quantity and the type of antibiotics), and standardizing prescribing practices across veterinary activities—critical steps that remain unfulfilled [14].

This narrative literature review was carried out using scientific databases, including Scopus, Web of Science, MDPI, and PubMed. The search strategy initially focused on key terms, such as “antimicrobial resistance”, “Europe”, “One Health”, “animals “and “companion animals”, but was progressively refined to include more specific terms related to the legislative framework, individual animal groups (e.g., aquaculture animals, farm animals, etc.), and bacterial resistant patterns as new themes emerged during the review process. Studies were selected based on their relevance to the topic, citation impact, and year of publication, with preference given to peer-reviewed articles published within the last five to ten years to ensure that the information reflects the most current knowledge and trends in the field. Highly cited studies were prioritized to capture influential and recognized research.

## 2. Antimicrobial Resistance

Antibiotics function in two primary ways: by killing bacteria or by inhibiting their proliferation [11,15]. Resistance to an antibiotic occurs when bacteria no longer respond to an antimicrobial agent that was originally intended to target and affect them [11,16].

When a bacterium is exposed to an antibiotic, susceptible cells are inhibited or killed, while those with pre-existing resistance genes survive and proliferate. This process is a natural aspect of developing drug resistance. Such selective pressure facilitates the spread of resistant traits within microbial populations. AMR is a natural and inevitable evolutionary process that predates the clinical use of antibiotics [8,11,15]. AMR refers to the ability of microorganisms to develop mechanisms that enable them to resist antimicrobial agents, such as antibiotics [3,16,17]. It can emerge through either intrinsic or acquired mechanisms. Intrinsic, or natural, AMR results from evolutionary processes, through spontaneous mutations or the acquisition of new genetic material, enabling microorganisms to adapt to their environmental conditions and be insensitive to an antibiotic without requiring additional resistance factors. Gram-negative bacteria, for instance, possess an outer membrane that naturally acts as a barrier [11,16]. Acquired AMR, on the other hand, develops in response to selective pressures, such as antimicrobial treatments [2,18]. Acquired resistance mechanisms refer to a bacterium that was originally susceptible to an antimicrobial compound and, through adaptive mechanisms such as chromosomal mutations or mobile resistance genes, like plasmids, phages, transposons, and other mobile elements capable of horizontal transmission between bacteria, becomes resistant to their action [11,16]. Horizontal gene transfer facilitates the rapid emergence of new bacterial phenotypes; for instance, conjugative plasmids can transfer antibiotic resistance genes across genera, orders, and even phyla [11,19]. A single organism can present several resistance mechanisms [20]. This transfer is particularly prevalent in high-bacterial-load environments, such as soil, animal gut microbiomes, wastewater treatment plants, and clinical settings, but it can also occur through food or companion animals [11,21,22].

Within a bacterial population, some individuals may carry antibiotic-resistant genes (ARGs). While these genes may not always confer a clinically significant level of resistance, they can reduce susceptibility, resulting in low-level resistance. This reduction in susceptibility is typically the first step towards the development of high-level resistance. ARGs can be transferred both vertically and horizontally [8]. Additional resistance mechanisms employed by bacteria include biofilm formation, which is observed in both Gram-positive and Gram-negative bacteria, and the production of persister cells. These persister cells, characterized by reduced metabolic activity, can tolerate antibiotics and resume growth once the stress is removed [11,23]. This accentuates the significant risks posed by the selection and spread of antimicrobial-resistant bacteria [11].

### Multidrug-Resistant Bacteria

Bacteria that show resistance to antimicrobials are a threat, and according to their resistance abilities, they can be classified as multidrug-resistant (MDR), extensively drug-resistant (XDR), or pan-resistant (PDR) bacteria. A multidrug-resistant bacterium is defined as lacking susceptibility to at least one antimicrobial agent in three or more drug categories. An extensively drug-resistant bacterium shows limited susceptibility to at least one agent in all but two or fewer antimicrobial categories, meaning it remains susceptible to only one or two categories. Pan-resistant bacteria are characterized by resistance to all agents across all antimicrobial categories [24,25,26,27,28]. MDR bacteria, such as *Staphylococcus aureus*, *Staphylococcus pseudointermedius*, and *Escherichia coli*, have already been documented in dogs, with organisms such as *Proteus mirabilis*, *Klebsiella pneumoniae*, *Staphylococcus* spp., and *E. coli* being reported as potentially extensively drug-resistant and pan-resistant bacteria in isolates collected from dogs [29].

## 3. Drug-Resistant Bacteria of Concern in Companion Animals

The use of antibiotics in both human and animal populations is a key driver of the emergence of antimicrobial resistance, enabling bacteria, both Gram-positive and Gram-negative, to develop the ability to withstand critical medicines [8]. Untreatable infections caused by MDR bacteria are now found in companion animals [30]. The levels of AMR in companion animals in Europe are already of great concern, as Table 1 demonstrates.

Several antimicrobial-resistant bacteria can be transmitted from pets to humans, posing a significant threat to public health. These include Gram-positive bacteria such as MRSA, methicillin-resistant Staphylococci, vancomycin-resistant enterococci, and Gram-negative bacteria like carbapenemase-producing *Enterobacteriaceae*, ESBL (extended-spectrum beta-lactamase), *E. coli*, *Pseudomonas* spp., and other Gram-negative bacteria [2,31,32]. Monitoring efforts should focus not only on these pathogenic bacteria but also on non-pathogenic species, as these species have the potential to acquire and disseminate resistance traits [2,33].

Several microorganisms are of particular concern due to their zoonotic risk, notably the ESKAPE pathogens. The ESKAPE acronym refers to a group of highly virulent, MDR bacteria, including *Enterococcus faecium*, *Staphylococcus aureus*, *Klebsiella pneumoniae*, *Acinetobacter baumannii*, *Pseudomonas aeruginosa*, Enterobacter species such as *Clostridium difficile*, *Proteus* spp., and pathogenic *E. coli*. These pathogens remain significant therapeutic challenges and are increasingly identified in companion animals [15,17]. Several studies have already shown transmission between pets and their owners [32,34,35,36].

### 3.1. Gram-Positive Bacteria of Concern in Companion Animals

#### 3.1.1. Methicillin-Resistant Staphylococci

MRSA. In Europe, the MRSA prevalence varies widely, ranging from less than 5% to over 25% in certain countries, with infections rising, particularly in Southern European countries [37].

*Staphylococcus aureus* is a significant pathogen associated with hospital- and community-acquired infections in humans, and while the incidence of hospital-acquired MRSA is declining, community-acquired MRSA infections are rising. Some strains, traditionally linked to skin infections, are now causing bloodstream infections in hospitals [28].

MRSA is particularly noteworthy due to its resistance to all β-lactam antimicrobials used in veterinary medicine and the fact that companion animals can act as reservoirs. This bacterium has been isolated from companion animals, causing conditions such as skin and soft-tissue infections, post-surgical wound infections, urinary tract infections, and pneumonia. Veterinary staff face an increased risk of MRSA colonization [31,38,39]. Transmission of MRSA from infected animals to their owners has already been reported [12].

MRSP. *Staphylococcus pseudointermedius* is the primary staphylococcal species that colonizes the skin and mucous membranes of cats and dogs, and it is an opportunistic human pathogen [40]. It is considered more virulent than MRSA in small animal species [12]. MRSP is a critical health concern in these animals. It causes infections in the skin, ear canal, and surgical sites, as well as conditions like hepatitis, gingivitis, urinary tract infections, peritonitis, respiratory infections, arthritis, and sepsis. Similar to MRSA, veterinary staff are at an increased risk of colonization by MRSP [31,39,41,42].

**Table 1:** Minimum and maximum percentage of AMR in microorganisms detected in companion animals in Europe. Data compiled from an EFSA (European Food Safety Authority) report on AMR in bacteria linked to transmissible diseases in cats and dogs. The bacterial species analysed included *S. pseudointermedius*, *S. aureus*, *E. coli*, *Proteus mirabilis*, *Klebsiella* spp., *Enterobacter* spp., and *P. aeruginosa*. Data were drawn from the literature and national surveillance systems (DANMAP, NORMVET, FINRES, SWEDRES-Svarm, RESAPATH, ANRESIS), with differences in the methods, interpretation criteria, and timeframes. Some results may lack significance due to the variability in the study design, sampling, and data availability across countries. Adapted from the [41].

	Gram-Positive		Gram-Negative				
Antibiotic	*S. pseudo*	*S. aureus*	*E. coli*	*Proteus* spp.	*Enterobacter* spp.	*Klebsiella* spp.	*P. aeruginosa*
3rd Cephalosporins ^1^	-	-	0.2–71	1.8–75	5.2–5.2	0–100	-
Aminopenicillins	-	-	12.1–100	9.1–28.9	-	100–100	-
Amox./Clav. ^2^	-	-	0–100	3.9–68.7	-	27.3–91.7	-
Fluoroquinolones	1–94.3	0–51.3	2–39.3	3.6–26.2	-	9.1–100	8–67.7
Nitrofurantoin	-	-	1–1.6	-	-	-	-
Sulfa./TMP ^3^	5–97.1	0–100	4.3–61.2	10.9–87.5	-	9.1–91.7	-
Fusidic Acid	6.1–38	-	-	-	-	-	-
Gentamicine	1.7–58.6	0–74.4	-	-	-	-	2–56.5
Lincosamides	13–98.6	4.4–100	-	-	-	-	-
Methicilin	0–41.4	0–35.9	-	-	-	-	-
Tetracyclines	20.2–95.7	10–60		-	-	-	-
Polymyxin B/Colistin	-	-		-	-	-	0–1

^1^ Third-generation cephalosporins. ^2^ Amoxicillin + Clavulanic Acid. ^3^ Sulphonamides + Trimethoprim.

#### 3.1.2. Other Species

*Enterococcus*. *Enterococcus faecalis* and *Enterococcus faecium* are clinically relevant species in humans, with vancomycin and ampicillin resistance documented in humans and dogs [28,31,41]. In animals, they are typically associated with ear infections and urinary tract infections. Although these bacteria are commensal organisms, they can acquire resistance and cause severe infections, including sepsis [31,41]. Both zoonotic transfer and fresh food have been considered reservoirs [28].

*Clostridium difficile*. It is often present in the gastrointestinal tract of cats and dogs, and also in other mammals, birds, and reptiles, generally without clinical manifestations [7]. It is naturally resistant to several antimicrobial agents [7]. Some hypervirulent strains can cause colitis and diarrhoea in humans, making companion animals such as cats, dogs, and horses a potential reservoir [31,39].

### 3.2. Gram-Negative Bacteria of Concern in Companion Animals

#### 3.2.1. *Enterobacteriaceae*

This family of Gram-negative bacteria includes several clinically significant species, such as *E. coli*, *Klebsiella* spp., *Proteus mirabilis*, *Enterobacter* spp., and *Salmonella* spp. Many of these microorganisms are part of the normal commensal flora of the gastrointestinal tract. Within this family, resistance mechanisms are often linked to the production of enzymes, including β-lactamases, carbapenemases, cephalosporinases, and plasmid-mediated AmpC β-lactamases [31,32,41,43,44]. Fluoroquinolone-resistant *Enterobacteriaceae* have also been identified [45].

*E. coli*. This pathogen is currently one of the most clinically significant agents affecting both humans and animals, and it must be recognized and treated as a critical public health threat [28]. It is the most prevalent Gram-negative bacterium isolated from blood and urine cultures, with sepsis being one of its most frequent and severe manifestations [28]. It is capable of acquiring resistance through horizontal gene transfer from other Enterobacterales [28]. In Europe, high resistance rates have been reported, particularly against aminopenicillins, fluoroquinolones, aminoglycosides, third-generation cephalosporins, and even carbapenems [28,31,44]. Numerous strains harbouring resistance genes have been detected in either commensal flora or clinical infections in both animals and humans [31].

As in humans, it is responsible for 50% to 60% of urinary tract infections diagnosed in companion animals, making it a primary etiological agent of these infections [41].

*Salmonella*. Several resistance mechanisms have been detected in isolates from companion animals, with reports of fluoroquinolones, β-lactamases, and cephalosporines resistance documented [31,39]. An MDR *Salmonella typhimurium* has been associated with gastrointestinal infections, both in companion animals and in humans [31].

Other Enterobacteriaceae. Some enzymes, like extended-spectrum β-lactamases and carbapenemases related to AMR, have been detected in *Citrobacter* spp. and *Klebsiella* spp. in both dogs and cats [31,46]. Across Europe, an increase in carbapenem-resistant *Klebsiella pneumoniae* was reported, and some hypervirulent strains are responsible for a high number of necrotizing fasciitis cases [28]. *Proteus mirabilis*, which is intrinsically resistant to antibiotics such as ampicillin, cefazolin, tetracyclines, and nitrofurantoin, is a common aetiological agent of otitis externa in dogs [41,47].

#### 3.2.2. Genus *Pseudomonas* and *Acinetobacter*

*Pseudomonas aeruginosa*. *P. aeruginosa* is an opportunistic pathogen responsible for nosocomial infections, severe infections in immunocompromised patients, and significant diseases in animals. Classified by the World Health Organization as a critical AMR priority pathogen [48], *P. aeruginosa* can form biofilms and exhibit intrinsic resistance to many antimicrobials, such as cephalosporines (except for ceftazidime), and trimethoprim/sulphamethoxazole, chronically persisting in the host and escaping antibiotic treatment, with documented resistance to last-resort polymyxin and carbapenem-class antibiotics [28]. In veterinary medicine, MDR is a common challenge when treating *P. aeruginosa* infections. Resistance to fluoroquinolones, amikacin, and gentamicin has also been documented [31,41]. *P. aeruginosa* is frequently associated with dermatological infections, such as otitis externa in dogs [41].

*Acinetobacter baumannii*. *A. baumannii* infections occur at lower rates when compared to other ESKAPE pathogens; however, it has higher MDR rates and the ability to rapidly develop resistance mechanisms, with as much as four times higher MDR levels than those observed in *P. aeruginosa* or *K. pneumoniae* [28]. Pan-drug-resistant isolates have been documented, with last-resort antibiotics such as carbapenem and polymyxin no longer proving effective [28]. *A. baumannii* can colonize the skin and oral cavity of dogs and has been linked to infections of the urinary tract and respiratory system, and to cases of sepsis. Notably, a carbapenem-resistant *A. baumannii* strain was identified in a urinary tract infection in a cat [31,49].

#### 3.2.3. Other Bacteria

*Campylobacter* spp. *Campylobacter* spp. are commonly found as part of the intestinal microbiota in dogs and can also be present in cats. Humans can become infected with *Campylobacter jejuni*, mainly by the ingestion of uncooked meat or contaminated water [7]. However, transmission between owners and pets has already been documented; dogs and cats have been identified as a significant risk factor for its transmission, mainly in urban areas [7,31].

*Bordetella bronchiseptica*. This respiratory pathogen can cause upper and lower respiratory infections in cats and dogs. It has documented resistance to amoxicillin–clavulanate and ampicillin [41]. It can be transmitted from pet to human. This agent has been associated with high mortality rates in immunocompromised infected people [50].

## 4. Antibiotic Classes and Safe Use Recommendations

The emergence of bacterial resistance to conventional antibiotics approved for veterinary use poses a serious and alarming threat to both animal and human health. Many antibiotics used in veterinary medicine are also critical for human medicine. Among the most commonly administered classes of antibiotics in animals are quinolones (primarily fluoroquinolones), aminopenicillins (both alone and in combination with potentiators), first- and second-generation cephalosporins, tetracyclines, sulphonamides (alone or potentiated), macrolides, glycopeptides, and third- and fourth-generation cephalosporins. All of these antibiotics are classified as the highest-priority critically important antibiotics (HPCIAs) for human health [8]. The absence of rapid diagnostic methods for identifying bacterial pathogens and AMR genes in clinical settings often leads to the overuse of broad-spectrum antibiotics [28].

The European Medicines Agency (EMA) has classified antibiotics into four categories based on their risk to public health and the necessity of their use in veterinary practice (Appendix A, Table A1). Respecting these guidelines in daily clinical practice is an absolute necessity while implementing a comprehensive approach to the AMR situation. The categories are as follows:

- Category A: Antibiotics in this category are reserved exclusively for human use and are not authorized for veterinary use in the European Union.
- Category B: This includes antibiotics considered critically important in human medicine. Their use in veterinary medicine is restricted and should only be considered as a last resort when all other options (from Categories C and D) have been exhausted.
- Category C: These antibiotics should be used with caution and only if those from Category D are ineffective.
- Category D: Representing the first-line treatment options in veterinary practice, these antibiotics should also be used judiciously to minimize resistance risks [51].

However, and according to European Legislation (articles 10/11 of Directive 2001/82/CE), in certain conditions and under veterinary responsibility, antibiotics deemed to human use alone can be use off-label in companion animals; carbapenems, mupirocin, nitrofurantoin, rifampicin, and vancomycin in restricted conditions and to avoid unacceptable suffering in animals [52].

## 5. Importance of Antibiotic Resistance—One Health Perspective

Each year, an estimated 5 million people die due to AMR, with 33,000 in European Union countries alone. This number is projected to rise to 10 million by 2050 [3,4,9,39,53]. Microorganisms are ubiquitous, and the interconnected triad of environments, humans, and animals, along with the bacterial gene flow between them, presents a significant challenge. Addressing this issue requires a coordinated and comprehensive approach [2]. Over the past 15 years, regulations regarding antibiotic use in animals have significantly increased due to the recognized impact on human health [8,11]. Many conventional antibiotics approved for veterinary use already show bacterial resistance, posing a serious threat to both human and animal health [8,14].

**Figure 1:**
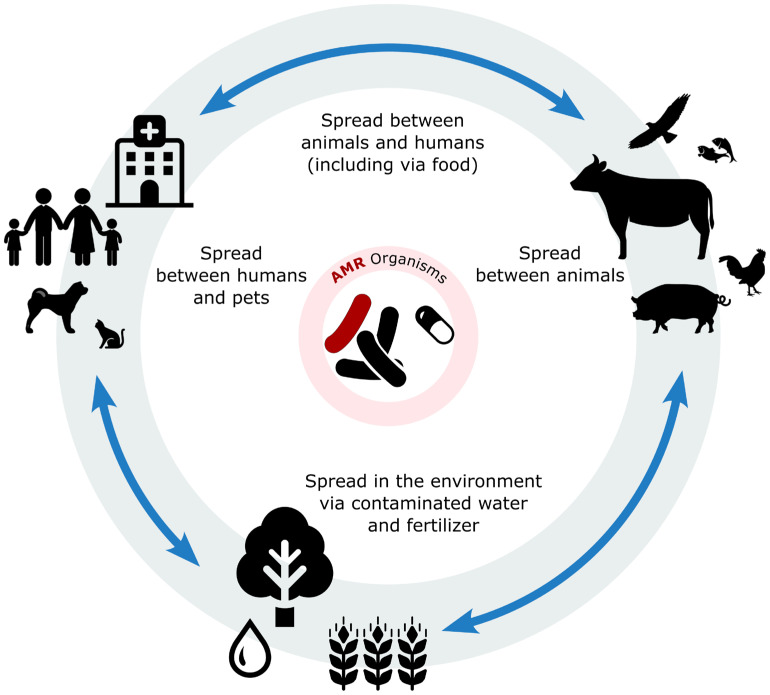
Illustration of the interconnected flow of antibiotic residues and bacteria among animals, humans, and the environment. Antibiotics are administered in both human and veterinary medicine contexts, including in companion animals and livestock production. Residual antibiotics and resistant bacteria can enter the environment through various routes, such as wastewater, manure application, and runoff. Once in the environment, these contaminants may infiltrate the soil and water systems, promoting the spread of resistant genes. Contaminated water and soil can reintroduce resistant bacteria into the food chain and water supply. Vectors and icons by SVG Repo www.svgrepo.com and https://openclipart.org/. accessed on 2 May 2025.

The presence of antibiotics in the environment is a significant concern as they are used in a wide range of scenarios beyond therapeutic settings. For instance, in aquaculture, antibiotics can persist for extended periods in both fish and the aquatic environment. In agriculture, streptomycin is commonly used in fruit and vegetable production. Additionally, in clinical settings, active antibiotic molecules are excreted by humans and animals through urine or faeces, contributing to environmental contamination [11,54,55,56]. There are numerous environmental reservoirs of AMR, collectively known as resistomes. These include solid and wastewater systems, animal and human healthcare facilities, wildlife habitats, and production animal systems (Figure 1) [17,53].

A study by Rodriguez-Mozaz et al. [56] reported the presence of 53 different antibiotics in the final wastewater effluent across seven European countries. This highlights that active antibiotic molecules, resistant bacteria, and mobile genetic elements persist in wastewater, enabling them to spread among bacterial populations. Consequently, both pathogenic and non-pathogenic bacteria may carry antimicrobial resistance genes [11]. Remarkably, wastewater treatment processes do not always eliminate residual antibiotics, and neither American nor European regulations currently impose limits on the concentrations of antimicrobial substances in wastewater [11].

The One Health concept, defined as the collaborative effort of multiple health science professions alongside related disciplines and institutions, which aims to achieve optimal health for people, domestic animals, wildlife, plants, and the environment at the local, national, and global levels, represents an integrated approach that is crucial [9,57]. It emphasizes the need for a comprehensive strategy to contain the spread of AMR, and as such, it needs to be implemented in every aspect, introducing effective measures to protect agricultural practice, the animal production and food chain, human and animal health, and wildlife and the environment [5,11]. Encouragingly, many developed countries have implemented measures to reduce antimicrobial use (AMU) in production animals without negatively impacting animal health or welfare. These efforts highlight the feasibility of reducing AMU while maintaining high standards of animal care [1,58,59]. The One Health approach acknowledges the deep connections and interdependence between animals, humans, and the environment (One Health Triangle) [11].

## 6. Animals’ Contribution to AMR

The AMR phenomenon affects humans and animals, extending between species and leading to economic losses and treatment failures [53,60]. The complexity of AMR in animals is far greater than in humans, necessitating a more cautious and comprehensive approach to antibiotic use. Antibiotics are commonly employed in production animals, such as cattle, poultry, swine, and aquaculture, for growth promotion, clinical treatment, and preventive measures. Additionally, they are extensively used in exotic and companion animals for both clinical and preventive care [8]. The specific role of companion animals in AMR will be discussed in detail in this paper.

### 6.1. Farm Animals

Intensive production systems are widely adopted to meet the growing demand for animal protein, but they facilitate the rapid spread of microorganisms among animals and their environment [11,54]. Antibiotics such as penicillins, tetracyclines, macrolides, quinolones, and aminoglycosides are routinely used in food-producing animals for treatment, prophylaxis, and growth promotion [2,8,33]. Prophylactic use, often via feed or water, aims to prevent infections related to respiratory diseases, liver abscesses, mastitis, and post-procedural complications [2,61], but it is criticized for driving antimicrobial resistance [11].

In 2006, the European Union banned antibiotics as growth promoters due to their role in selecting resistant bacteria [2,53]. However, at least 40 countries, including the United States of America, still permit this practice [8,11]. The widespread use of antibiotics in livestock leads to environmental contamination via manure (often used as fertilizer) and wastewater (used in irrigation), posing risks to wildlife, human health, and food safety, as residues are detected in meat, milk, and eggs, making the food chain a critical pathway for AMR transmission from animals to humans [8,62]. Resistant bacteria, such as *Campylobacter* spp., *Salmonella* spp., *Staphylococcus* spp., and *Enterococcus* spp., present in food-producing animals further exacerbate the risk of AMR transmission [8].

Global antibiotic use in food animals was estimated at 63,000 tons in 2010, with projections reaching 105,000 tons by 2030 [63]. Therapeutic administration often involves entire livestock groups, significantly contributing to overuse [54]. Nonetheless, many developed nations have successfully reduced antimicrobial use (AMU) without compromising animal health or welfare [1,59,64]. Since 1969, the Swann Committee in Europe has advocated for distinct antibiotic regulations for human and veterinary use; however, this approach remains inconsistently applied, particularly in the U.S. and Canada [11].

### 6.2. Aquatic Environment and Aquaculture

The aquatic environment is a major waste recipient, including antibiotics excreted unchanged or metabolized through faeces and urine from humans and animals. These residues exert selective pressure on aquatic ecosystems, fostering the development of AMR in bacteria. Additionally, such residues can accumulate and magnify within the aquatic food chain, further exacerbating the impact of AMR [8,65,66]. Pathogens originating from aquatic environments often show high levels of resistance [67,68]. Antibiotic-resistant bacteria and antibiotic-resistant genes (ARGs) have been documented in both tap and bottled water, highlighting the pervasive nature of this issue [69].

Aquaculture, an intensive food production system, accounts for nearly half of the fish and fish by-products consumed globally. Antibiotics used in aquaculture, whether for therapeutic or nontherapeutic purposes, contribute significantly to environmental contamination, with approximately 80% of these antibiotics entering the environment in their active form [8,70]. Commonly used classes of antibiotics in aquaculture include sulphonamides, phenicols, penicillins, quinolones, and tetracyclines. Their widespread use facilitates the spread of AMR among aquatic organisms, with potential transmission to humans and other species [2,71,72]. A notable concern arises from the use of aquaculture waste products, such as salmon sludge (comprising uneaten food and faeces), as fertilizers. This practice poses a significant risk of further dissemination of AMR, as these waste products may carry antibiotic residues and resistant bacteria into terrestrial ecosystems, amplifying the spread of resistance [8,73].

### 6.3. Exotic Animals and Wildlife

Unfortunately, there is a lack of data regarding AMR in exotic animals [48]. Furthermore, no guidelines, action plans, or recommendations currently exist for the prudent and responsible use of antibiotics in exotic species [8,48,74]. The practice of exotic animal medicine is particularly challenging due to the limited availability of antimicrobials. This limitation is primarily due to the potential for toxic or adverse effects in various exotic species and issues related to product formulation, which effectively reduces the range of treatment options [74]. Consequently, antimicrobial prescriptions are often empirically based, aiming to cover all suspected pathogens. This approach frequently results in the off-label use of critically important antibiotics, raising significant concerns about AMR development. Several MDR bacteria have already been described in ornamental bird species, rabbits, other mammals such as guinea pigs and chinchillas, and turtles [48,74,75,76,77]. Rabbits are common pets in several European countries and can host zoonotic bacteria, such as *Pasteurella* spp. and *Bartonella* spp. Given the frequent practice of empirical antibiotic prescription, data on antimicrobial resistance AMR in these species are essential to optimize antibiotic use [78]. The emergence of antimicrobial-resistant strains like *Pseudomonas aeruginosa*, *Acinetobacter baumannii*, or *Klebsiella pneumoniae* in pet rabbits poses a health risk to their owners, as these opportunistic pathogens are major contributors to hospital mortality worldwide and are classified as critical threats by the Centres for Disease Control [78].

Exposure to AMR bacteria and resistance genes in wildlife is a growing concern, as environmental contamination of soil and water with antibiotic residues and antibiotic-resistant microorganisms is common [79,80,81]. This contamination facilitates the transmission of AMR to wildlife species, further contributing to the global AMR crisis [8]. Several bird species can function as reservoirs of AMR bacteria and AMR genes [82].

### 6.4. Urban and Racing Pigeons

Racing pigeons and free-living pigeons play a significant role in public health due to their proximity to humans and their potential to act as reservoirs of pathogenic microorganisms [83]. Racing pigeons are bred and housed near human dwellings and participate in national and international races where they are transported to distant locations and released to navigate their way home. Free-living pigeons, on the other hand, inhabit rural and urban areas, including historical sites, parks, and public squares, maintaining close contact with humans in both settings [83].

These birds have been identified as reservoirs of several zoonotic pathogens, including *Chlamydia psittaci* (chlamydiosis), *Cryptococcus* spp. (cryptococcosis), and *Aspergillus* spp. (aspergillosis). Additionally, they are carriers of antimicrobial-resistant bacteria, such as MRSA, *Campylobacter* spp., *Salmonella* spp., *Enterococcus* spp., and multidrug-resistant *E. coli* [83,84,85,86,87]. Studies have revealed bacterial isolates from pigeons that are resistant to at least three antimicrobial classes, classifying them as multidrug-resistant pathogens [86]. This issue is compounded by the lack of regulations in this domain, with pigeons freely crossing city and national borders, potentially disseminating antimicrobial-resistant microorganisms along their routes. This highlights the urgent need for better regulation and oversight of antimicrobial use in racing pigeons as well as for more comprehensive studies on their role in the dissemination of AMR. The widespread use of antibiotics among pigeon breeders allows for antibiotics to be administered without veterinary oversight, using inappropriate doses, durations, or even when bacterial infection is not present [86,88]. Some breeders purchase multidrug mixtures from unregulated sources, combining antibiotics from various classes, some of which are intended for human use. These mixtures are often shared among breeders, further compounding the problem [86,87].

### 6.5. Companion Animals

The emergence and spread of AMR in companion animals, especially cats and dogs, pose a substantial One Health challenge, given the close interdependence of human, animal, and environmental health. Several MDR pathogens commonly associated with pets, including MRSA, MRSP, VRE, ESBL-producing *E. coli*, *K. pneumoniae*, and carbapenemase-producing Enterobacteriaceae, represent a growing public health threat [31,89,90,91]. A study by Muñoz-Ibarra et al. identified *Pseudomonas* spp. and *Enterococcus* spp. as the bacteria with the highest levels of AMR among dogs, cats, and exotic pets [48].

Companion animals are frequently exposed to, and serve as reservoirs of, antibiotic-resistant bacteria (ARB), ARGs, and MDR organisms [92,93,94]. Among these, *E. coli* is of particular concern. It is a common commensal organism in animals and a leading cause of digestive, urogenital, and occasionally renal infections in cats and dogs. *E. coli* isolates from pets have demonstrated resistance to a range of antimicrobials, with up to 27% exhibiting resistance to at least one agent, making it a reliable indicator of the selective pressure exerted by antimicrobial use (AMU) [1,51].

Human–animal interactions, including licking, petting, and shared living environments, promote bidirectional transmission of resistant microorganisms. ARB and ARGs are transmitted through direct contact with saliva, faeces, urine, and aerosols, as well as via contaminated environmental surfaces [17,95,96]. Bacterial urinary tract infections, in companion animals, for example, are a significant public health concern due to the frequent involvement of MDR, XDR, and PDR bacteria, many of which are potentially zoonotic; these agents are a therapeutic challenge and a health hazard [47].

Moreover, ARGs present in non-pathogenic bacteria may be horizontally transferred to pathogenic strains, further compounding resistance risks [97]. Importantly, human-to-animal transmission has also been documented, as in the case of MRSA, which now circulates bidirectionally between people and pets [43,98].

Antibiotic consumption patterns in companion animals mirror those in humans. Over 70% of veterinary prescriptions involve broad-spectrum antibiotics and critically important antimicrobials (CIAs), with amoxicillin–clavulanate being the most frequently used [1,11]. Particularly concerning is the widespread use of CIAs vital for human medicine, including fluoroquinolones, macrolides, and third- and fourth-generation cephalosporins, which significantly increases the potential for resistance development and cross-species dissemination [1,17,96,99,100,101,102,103].

Recent reports of colistin resistance in pets in some European countries further highlight the gravity of the situation [1]. Polymyxin B, a related agent, is commonly used in topical veterinary treatments, such as for otitis. Given their classification as last-resort antimicrobials in human medicine, the use of such agents in animals should be stringently restricted and never prioritized as first-line therapy [1,104]. A study by Garcias et al. demonstrated a rising trend in AMR, in almost all antimicrobial classes, among agents causing otitis externa in the Iberian Peninsula between 2010 and 2021, particularly in Pseudomonas and Enterococcus species [105].

Several pet-associated pathogens have demonstrated resistance not only to first-line agents but also to critical last-resort antibiotics, including ampicillin, imipenem, colistin, methicillin, cefotaxime, lincomycin, fluoroquinolones, cephalosporins, and vancomycin [17]. Inappropriate and extra-label antibiotic use in pets, often without robust pharmacological guidance, exacerbates these risks by contributing to suboptimal dosing, reduced efficacy, and increased resistance selection pressure [8,106]. Empirical antimicrobial therapy in companion animals is increasingly challenged by the rising prevalence of AMR bacteria, like MDR *Enterococcus* spp., *Enterobacter* spp., *P. aeruginosa*, and *K. pneumoniae*, and the disseminated resistance to several antimicrobial classes (aminoglycosides, fluoroquinolones, and carbapenems) [40]

Veterinary facilities play a significant role in ARB dissemination. Environmental reservoirs in clinics and hospitals, including drains, scales, holding areas, transport gurneys, high-contact surfaces, and exam rooms, pose a risk to both animals and human staff. Infections acquired in these settings mirror human hospital-acquired infections in their severity and potential for spread. Occupational exposure among veterinary personnel further amplifies these risks [9,107,108].

Although this review centres on cats and dogs, other species, including horses, rabbits, birds, rodents, fish, and reptiles, are also considered companion animals. Their close contact with humans, combined with better veterinary care, suggests their potential involvement in AMR transmission dynamics. Despite the growing body of evidence linking companion animals to AMR risks, specific regulatory guidance from the European Medicines Agency (EMA) remains notably absent.

## 7. European Situation

In Europe, over 79 million households own a cat, while 68 million have a dog [6]. According to the EFSA, *S. pseudointermedius*, *E. coli*, and *P. aeruginosa* were identified as the most relevant bacteria with AMR in the European Union [41]. Currently, there is no mandatory requirement for European countries to report AMU in companion animal practices. However, this measure is expected to become mandatory by 2030 at the latest [109]. Within the European Union, ARB accounts for up to 20% of human infections, with Greece reporting an alarming 40% [110]. The most prevalent bacterial species found in nosocomial and community infections in the human species are *E. coli*, *S. aureus*, and *P. aeruginosa*, followed by *K. pneumoniae*, *Enterococcus* spp., *P. mirabilis*, and *Enterobacter* spp. The most prevalent bacteria found in cats and dogs are *Staphylococcus* spp., *Streptococcus* spp., *Pseudomonas* spp., *E. coli*, and *Enterococcus* spp., with *Enterococcus* spp. and *Pseudomonas* spp. exhibiting the highest levels of AMR [40]. This highlights the urgent need to control AMU and AMR in both human and animal populations. Several surveillance agencies and networks across Europe are involved in monitoring AMR and AMU in humans, animals, and the food chain to address this growing public health concern.

- European Antimicrobial Resistance Surveillance Network (EARS-Net): This network collects data on eight pathogens of critical importance to human health, including *Streptococcus pneumoniae*, *S. aureus*, *E. faecalis*, *E. faecium*, *E. coli*, *Klebsiella pneumoniae*, *P. aeruginosa*, and *Acinetobacter* spp.
- European Surveillance of Antimicrobial Consumption Network (ESAC-Net): The ESAC-Net focuses on gathering data related to antimicrobial consumption in humans, enabling an understanding of usage patterns across European countries.
- European Food and Waterborne Diseases and Zoonoses Network (FWD-Net): This network collects AMR data on foodborne pathogens, specifically *Salmonella* spp. and *Campylobacter* spp., which pose significant risks to public health.
- European Food Safety Authority (EFSA): The EFSA conducts harmonized monitoring of AMR in zoonotic and commensal bacteria from food-producing animals. This includes *Salmonella* spp., *Campylobacter* spp., *E. coli*, and *Enterococcus* spp.
- European Surveillance of Veterinary Antimicrobial Consumption (ESVAC): ESVAC monitors antimicrobial usage in animals, providing critical insights into AMU trends in veterinary medicine.
- Global Antimicrobial Resistance Surveillance System (GLASS): Operated by the WHO (World Health Organization), the GLASS gathers global data on resistance patterns among human-priority bacterial pathogens.

These networks and organizations play an integral role in implementing the One Health approach to AMR surveillance by linking human, animal, and environmental health data. Their collective efforts provide critical information to guide policy development and risk mitigation strategies across Europe [111]. However, these surveillance organizations still lack effective measures for environmental data collection [111]. Most of these programmes are focused on production animals (that have achieved a significant reduction in antibiotic use without negative effects on their health and profits). A similar effort should now be directed towards companion animals [51,112].

Integrating existing agencies and programmes could enhance their effectiveness by streamlining data collection and analysis. A unified approach would improve consistency, reproducibility, and accessibility, facilitating more comprehensive AMR and AMU monitoring. This consolidation could lead to more efficient policymaking and better-informed strategies to combat antimicrobial resistance.

**Figure A1:**
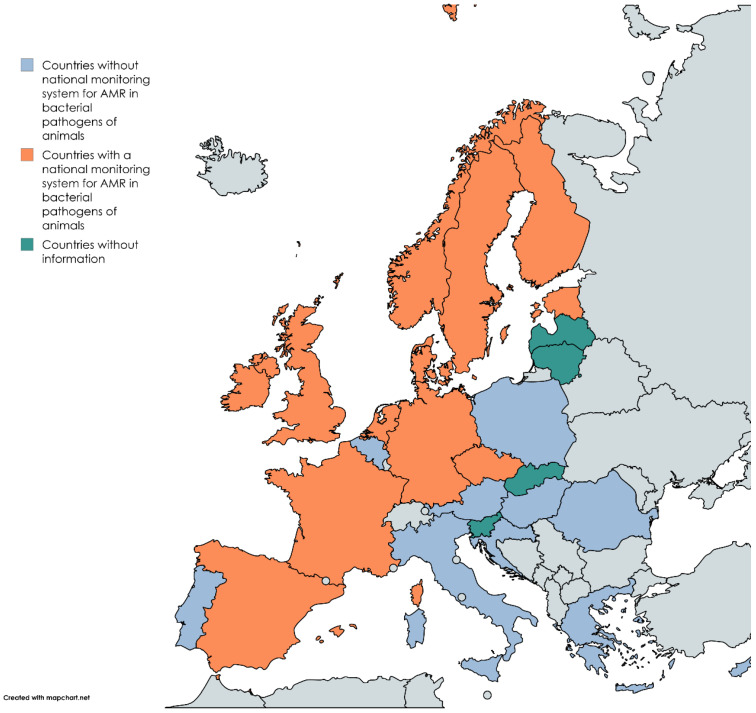
Map of European countries affiliated with the European Union with at least one national monitoring system for AMR for bacterial pathogens of animals, without any surveillance system, and without information. Adapted from EU-JAMRA and reference [114]. Made in mapchart.net.

Regarding AMR control, existing European legislation focuses exclusively on food-producing animals and no active monitoring systems are in place for companion animals [112]. Moreover, antibiotic use in companion animals is absent from the EMA annual reports, primarily due to the lack of data on cat and dog populations in most European countries. Only a limited number of European nations actively report on AMR in companion animals (Appendix A Figure A1) [41]. Efforts to estimate the antibiotic use in companion animals, such as those conducted in Nordic European countries, rely on sales data. However, this approach does not provide critical insights into the clinical context or the appropriateness of antibiotic use. To improve antimicrobial stewardship and enhance the understanding of AMU in companion animals, a comprehensive and standardized control programme must be developed and implemented across all European countries. This would enable the collection of accurate data on antibiotic use and its impact on resistance in companion animal therapies [109].

In the treatment of cats and dogs, the most commonly used antimicrobials in countries such as Denmark, Finland, Italy, Sweden, Norway, and the United Kingdom are β-lactams, such as amoxicillin and amoxicillin combined with clavulanic acid. Other frequently used antibiotics include cephalosporins, macrolides, fluoroquinolones, tetracyclines, nitroimidazoles, and trimethoprim/sulphonamides [30,33]. Since 2014, the Netherlands has implemented stricter regulations on the use of certain CIAs; for instance, antimicrobial sensitivity testing is now mandatory before administering third-generation cephalosporins in animals. This policy represents an important step towards promoting the responsible use of antibiotics and reducing the risk of antimicrobial resistance [1]. A review of existing monitoring programmes revealed that several countries, including the Czech Republic, Denmark, Estonia, Finland, Germany, Ireland, France, Norway, Sweden, and the Netherlands, have already operational monitoring and surveillance programmes for AMR and AMU. Meanwhile, countries like Spain, Belgium, and Greece are currently developing their control programmes [51].

Regarding veterinary health, the European Union plans to establish the European Antimicrobial Resistance Surveillance Network in Veterinary Medicine (EARS-Vet) to assess the current state of AMR and implement comprehensive AMR control programmes [51]. The EARS-Vet aims to monitor six key animal species—cats, cattle, chickens (both layers and broilers), dogs, swine, and turkeys—as well as 11 bacterial species: *E. coli*, *Actinobacillus pleuropneumoniae*, *K. pneumoniae*, *Pasteurella multocida*, *S. aureus*, *Mannheimia haemolytica*, *S. pseudointermedius*, *Staphylococcus hyicus*, *Streptococcus suis*, *Streptococcus uberis*, and *Streptococcus dysgalactae*. The establishment of the EARS-Vet will serve as a significant step forward in creating standardized, harmonized data collection protocols regarding veterinary practice across member states, enabling more effective AMR control and fostering the One Health approach to address AMR comprehensively [51].

## 8. Veterinarians and Their Role in Combating AMR

The present necessity of reducing antibiotic use in animals puts a great emphasis on veterinary action. A study revealed a significant connection between preventive healthcare measures, such as vaccination, insurance, and neutering, and reduced antibiotic usage. This finding emphasizes the importance of adopting a comprehensive veterinary approach to health management [113]. Veterinarians play a critical role in ensuring the responsible and judicious use of antibiotics. Before prescribing, they should conduct a thorough clinical evaluation, limit antibiotic use in food-producing animals, avoid off-label applications, and determine dosage regimens based on scientific evidence. CIAs should only be used in exceptional cases, such as when susceptibility testing supports their necessity or in life-threatening conditions. All prescriptions must align with the principles of responsible antimicrobial stewardship [8]. To reduce the risk of resistance, veterinarians should prioritize antibiotics not used in human medicine and those with narrow spectra. Maintaining the efficacy of existing antimicrobials depends on their prudent use. In companion animal medicine, the primary concern is not the quantity of antibiotics used but rather their appropriateness and quality [1,104].

Professional organizations have issued guidelines to promote the correct use of antibiotics, such as those developed by the Federation of European Companion Animal Veterinary Associations (FECAVA) and the British Small Animal Veterinary Association (BSAVA). The implementation of such guidelines is a crucial step in optimizing antibiotic use and advancing antimicrobial stewardship [114]. These guidelines align with the definition and core principles of antimicrobial stewardship, as outlined by the American Veterinary Medical Association (AVMA) [113]. Recent studies, however, indicate that veterinary general practitioners remain reluctant to fully adhere to antibiotic prescribing guidelines [115]. Their decisions are influenced by the clinical presentation, personal experience, fear of treatment failure, self-confidence, drug availability, access to alternative therapies, and awareness of their role in public and animal health [113]. Understanding the risks of AMR in companion animals also impacts prescribing behaviour [8]. Practical factors such as the drug formulation, bacterial culture results, ease of administration, financial constraints, client expectations, and economic incentives further shape prescribing patterns [8,113]. Despite increased awareness, many veterinarians continue to rely on traditional prescribing habits rather than evidence-based guidelines [31,113]. Additionally, prescribing errors, whether due to intentional deviation or inadvertent mistakes, such as incorrect dosages or inappropriate drug selection, contribute to antibiotic misuse [11].

## 9. Future Endeavours

Antibiotic resistance is now considered an emergency, and a post-antibiotic era is increasingly seen as a possibility. Studies depict a gloomy future in this regard [2]. Antibiotic resistance originating from animals significantly contributes to the global AMR crisis, highlighting the urgent need for decisive measures to address this issue [2,31]. In 2015, the Global Action Plan on Antimicrobial Resistance (AMR-GAP) was developed, followed by the implementation of National Action Plans (AMR-NAPs) in various countries [4,116]. These strategies aim to mitigate AMR through a comprehensive set of measures, including the following:

- Surveillance systems: Continuous and systematic collection, analysis, and interpretation of health-related data to plan, implement, and evaluate public health practices.
- Stewardship programmes: Regulation of antimicrobial use to preserve its effectiveness and ensure availability.
- Pharmaceutical policies: Limiting antibiotic use without a valid prescription.
- Information campaign: Raising awareness of AMR and responsible antibiotic use.
- Infection prevention and control programmes: Strategies to prevent infections and limit the spread of resistance.
- Vaccination encouragement: Promoting vaccination to reduce reliance on antibiotics.

Such measures enable authorities to assess the scale of AMR, identify emerging resistance, and monitor the spread of specific resistances, including those involved in outbreaks [11,111]. The effective implementation of such measures is an ongoing effort that should be a top priority.

In adopting a One Health approach, veterinarians and physicians should collaborate to optimize, rationalize, and promote the prudent use of antimicrobial therapies across domestic, companion, and exotic animals, as well as humans, recognizing that bacterial pathogens and their resistance mechanisms can be shared between animals, humans, and the environment [48].

In addition to restricting and improving antibiotic prescription practices, it is crucial to enforce prophylactic interventions. Measures such as vaccination and the use of probiotics, symbiotics, postbiotics, and prebiotics, which directly influence the microbiota, play a vital role. An optimized microbiota enhances the immune system, improves the feed conversion efficiency, and increases resistance to pathogens [2,117,118]. Additionally, advanced therapies like faecal microbiota transplantation and bacteriophage therapy are viable alternatives [19,51,119]. The use of bioactive peptides, such as Nisin A, with bacteriostatic or bactericidal properties represents another preventive approach [2,19,120]. Predatory bacteria have also been proposed as a potential antimicrobial alternative; however, the lack of specificity between commensal and pathogenic bacteria presents a significant risk that warrants further investigation [2].

The risk of horizontal gene transfer of AMR between pets and their owners constitutes an important concern, as resistant genes can spread between both species, emphasizing that further and comprehensive studies on this matter are a priority in developing an effective global surveillance system [105].

Innovations, such as the development of rapid diagnostic tests able to accelerate bacterial infection detection and the precise identification of antibiotic resistance profiles, enhancing One Health monitoring through metagenomics in AMR surveillance (metagenomics, according to the National Human Research Institute, is the study of the structure and function of entire nucleotide sequences isolated and analysed from all the organisms, typically microbes, in a bulk sample), and genome-based research that allows the identification of novel drug targets to support the development of new antimicrobial agents and therapies, are an absolute necessity [13]. Along with research on new antimicrobial classes and viable alternatives, structural and comprehensive legislative measures and enforcement policies are vital to ensure a meaningful change and protect public health [2].

## 10. Discussion

Despite growing efforts to address AMR in Europe, significant knowledge and structural gaps continue to hinder progress, particularly in the context of companion animals. Current surveillance systems, such as the EARS-Vet, represent important steps forward; however, their effectiveness is limited by methodological inconsistencies, fragmented data collection, low population representativeness, and restricted data access [1,121]. Furthermore, over 75% of national AMR action plans in Europe remain unfunded, delaying implementation and weakening coordinated responses [122].

The lack of harmonized surveillance frameworks and standard protocols, especially for critical resistance mechanisms such as carbapenem resistance, impairs the comparability of data and the accuracy of risk assessments across regions [43,121]. Additionally, AMR surveillance in veterinary medicine is constrained by passive reporting systems, under-representation of companion animal isolates, and absence of centralized data integration. Advanced tools such as microsimulation modelling and machine learning offer potential to improve predictive analytics and inform evidence-based policies, but they remain underutilized [122].

In veterinary contexts, key non-antimicrobial risk factors associated with AMR, such as the ones described in humans, hospitalization durations, surgical interventions, and comorbidities, are insufficiently explored [31]. Understanding these broader determinants, alongside patterns of antimicrobial use, is critical to developing effective stewardship strategies. The rise of multidrug-resistant Gram-negative bacteria further underscores the urgent need for new antimicrobial development in both human and veterinary medicine [31].

Addressing AMR requires a coordinated, multi-sectoral strategy rooted in the One Health approach. This involves aligning public health, veterinary, and environmental sectors through integrated policies, standardized surveillance systems, and cross-sectoral collaboration. Improving AMR monitoring, expanding the use of rapid diagnostics, and strengthening regulatory frameworks to ensure responsible prescribing are essential components. Preventive strategies, including vaccination, enhanced biosecurity, and microbiota-targeted interventions (e.g., probiotics, prebiotics, postbiotics), can further reduce antimicrobial reliance [2,117,118].

Public awareness and education are vital to driving behavioural change and supporting stewardship at the community level [13]. In parallel, robust surveillance of antimicrobial residues in humans, animals, food, and the environment must be implemented across Europe. A centralized registry system would allow for more comprehensive tracking of resistance patterns, identify gaps in current practices, and guide targeted interventions [64]. In developing countries, AMR remains under-researched, with the risk factors including a high prevalence of stray animals and weak enforcement of health regulations for animals and humans [16].

Veterinary prescribing practices, in particular, demand urgent reform. Evidence-based guidelines, continuous professional training, and harmonized EU regulations are needed to support veterinarians in optimizing antibiotic use [113]. Although regulatory actions, such as the ban on antibiotic growth promoters, represent important progress, a globally coordinated response remains absent [2,123]. Alarmingly, antimicrobial consumption continues to rise in both the human and animal sectors despite surveillance efforts, emphasizing the urgent need for more effective monitoring and policy enforcement [1,11,109].

## 11. Conclusions

AMR is a growing global health crisis, recognized as a silent pandemic that severely compromises the treatment of bacterial infections in both humans and animals [51,67]. Despite legislative efforts in Europe, such as Regulation (EU) 2019/6, persistent challenges, including poor guideline adherence, fragmented surveillance, unrestricted antibiotic access, and limited public awareness, continue to hinder effective AMR control [109,124].

A One Health approach is essential to address AMR, integrating infection prevention, antimicrobial stewardship, regulatory enforcement, surveillance improvements, and public engagement. While global frameworks like the Global Action Plan (GAP) have driven policy progress [116], the gaps in surveillance and data comparability remain significant barriers [37,96]. Surveillance systems must evolve, incorporating genomic sequencing, expanded testing, and digital health tools to generate robust evidence for policymaking [122].

Implementing coordinated One Health interventions—including prudent antimicrobial use in veterinary practice, enhanced diagnostics, vaccination, and biosecurity—can prevent over 600,000 infections, save thousands of lives, and yield billions in healthcare savings and productivity improvements [125]. Without urgent action, resistance to last-resort antibiotics may triple by 2035, threatening both modern medicine and global health security [125]. Sustained investment in AMR mitigation is therefore critical to protect human and animal health, ecosystems, and economies.

## Data Availability

No new data were created or analysed in this study. Data sharing is no applicable to this article.

## Abbreviations

The following abbreviations are used in this manuscript:

AMR-NAP	Antimicrobial Resistance National Action Plan
AMR	Antimicrobial Resistance
AMR-GAP	Antimicrobial Resistance Global Action Plan
AMU	Antimicrobial Use
ARB	Antibiotic-Resistant Bacteria
ARG	Antibiotic-Resistant Gene
AVMA	American Veterinary Medical Association
BSAVA	British Small Animal Veterinary Association
CIA	Critically Important Antibiotics
EARS-Net	European Antimicrobial Resistance Surveillance Network
EARS-Vet	European Antimicrobial Resistance Surveillance Network in Veterinary Medicine
EFSA	European Food Safety Authority
EMA	European Medicines Agency
ESAC-Net	European Surveillance of Antimicrobial Consumption Network
ESBL	Extended-Spectrum Beta Lactamase
ESKAPE	*Enterococcus faecium*, *S. aureus*, *Klebsiella pneumoniae*, *A. baumannii*, *P. aeruginosa*, *Enterobacter*
ESVAC	European Surveillance of Veterinary Antimicrobial Consumption
EU	European Union
FECAVA	Federation of European Companion Animal Veterinary Association
FWD-Net	European Food and Waterborne Diseases and Zoonoses Network
GAP	Global Action Plan
GLASS	Global Antimicrobial Resistance Surveillance System
HPCIA	Highest-Priority Critically Important Antibiotics
MDR	Multidrug Resistant
MRSA	Methicillin-Resistant *S. aureus*
MRSP	Methicillin-Resistant *S. pseudointermedius*
US	United States of America
VER	Vancomycin-Resistant *Enterococcus*
XDR	Extensively Drug Resistant

## Appendix A

**Table A1:** Classification of antibiotics by category (EMA categorization of antibiotics for use in animals).

Category A—AVOID
Aminopenicillins
Ketolides
Monobactams
Rifamycins (except rifaximin)
Carboxypenicillin and ureidopenicillin (including combinations with lactamase inhibitors)
Carbapenems
Lipopeptides
Oxazolidinones
Riminophenazines
Sulfones
Streptogramins
Drugs used solely for tuberculosis and mycobacterial diseases
Other cephalosporins and penems (including 3rd-generation cephalosporins with β-lactamase inhibitors)
Glycopeptides
Glycylcyclines
Phosphonic acid and derivates
Pseudomonic acids
Substances newly authorized in human medicine
Category B—Restrict
Cephalosporins 3rd and 4th generation, except combinations with β-lactamase inhibitors
Polymyxins
Quinolones (fluoroquinolones and others)
Category C—Caution
Aminoglycosides (except spectinomycin)
Aminopenicillins in combination with β-lactamase inhibitors
Cephalosporins 1st and 2nd generation and cephamycins
Amphenicols
Lincosamides
Pleuromutilins
Macrolides
Rifamycins (only rifaximin)
Category D—Prudence
Aminopenicillins, without β-lactamase inhibitors
Tetracyclines
Natural narrow-spectrum penicillins (β-lactamase-sensitive penicillins)
Aminoglycosides (only spectinomycin)
Anti-staphylococcal penicillins (β-lactamase-resistant penicillins)
Sulphonamides, dihydrofolate reductase inhibitors, and combinations
Cyclic peptides
Nitroimidazoles
Steroid antibacterials
Nitrofuran derivates
